# Thrombospondin-1: A Key Protein That Induces Fibrosis in Diabetic Complications

**DOI:** 10.1155/2020/8043135

**Published:** 2020-06-11

**Authors:** Linhao Xu, Yong Zhang, Jian Chen, Yizhou Xu

**Affiliations:** ^1^Department of Cardiology, Affiliated Hangzhou First People's Hospital, Zhejiang University School of Medicine, 310006 Zhejiang, China; ^2^School of Basic Medical Sciences & Forensic Medicine, Hangzhou Medical College, Hangzhou, 310053 Zhejiang, China; ^3^Translational Medicine Research Center, Affiliated Hangzhou First People's Hospital, Zhejiang University School of Medicine, Hangzhou, 310006 Zhejiang, China; ^4^Department of Urology, The Second Affiliated Hospital, School of Medicine, Zhejiang University, Hangzhou, 310009 Zhejiang, China

## Abstract

Fibrosis accompanies most common pathophysiological features of diabetes complications in different organs. It is characterized by an excessive accumulation of extracellular matrix (ECM) components, the response to which contributes to inevitable organ injury. The extracellular protein thrombospondin-1 (TSP-1), a kind of extracellular glycoprotein, is upregulated by the increased activity of some transcription factors and results in fibrosis by activating multiple pathways in diabetes. The results of studies from our team and other colleagues indicate that TSP-1 is associated with the pathological process leading to diabetic complications and is considered to be the most important factor in fibrosis. This review summarizes the molecular mechanism of increased TSP-1 induced by hyperglycemia and the role of TSP-1 in fibrosis during the development of diabetes complications.

## 1. Introduction

Diabetes mellitus (DM) is one of the major metabolic and epidemic diseases that have increased significantly both in developed and developing nations. For instance, the prevalence of diabetes was less than 1% in the Chinese population in 1980, but in 2013, it affected approximately 11.6% of the adult Chinese population [1]. Based on their different etiologies, two types of diabetes cases are recognized. Although the underlying mechanisms of pathogenesis and the number of people affected are not the same for type 1 diabetes mellitus (T1DM) and type 2 diabetes mellitus (T2DM), some chronic complications induced by hyperglycemia are shared by people with either of these two distinct types of diabetes [2–4]. Due to these complications, diabetic patients experience significant morbidity and mortality [4, 5].

Diabetic complications pose a risk for metabolic abnormalities, such as endothelial dysfunction, inflammation, vasoconstriction, oxidation, and fibrosis. Among these results of diabetes, fibrosis, the chronic complications of which are features of both distinct types of diabetes, is an important determinant of morbidity and mortality for diabetic patients [2, 4]. Fibrosis can occur in various organs and tissues of diabetes patients, including the heart, liver, kidney, and retina [6–8]. Furthermore, intensified fibrosis and endothelial dysfunction in the penile corpora cavernous were diagnosed in streptozotocin- (STZ-) induced diabetic rats and resulted in erectile dysfunction (ED), one of the most common penile symptoms of diabetes mellitus [9]. Although the complete mechanism for fibrosis progression in diabetes remains unclear, glucose metabolism and insulin resistance are considered the major forms of fibrosis etiopathogenesis.

The process of fibrosis requires the dynamic participation of the extracellular matrix (ECM) and is controlled by a family of structurally unrelated macromolecules called multicellular proteins [4, 10]. Mounting evidence indicates that thrombospondin-1 (TSP-1), as one kind of glycoprotein, plays an important role in vascular remodeling by regulating the arterial response to injury and is a protein found widely in the ECM [11]. Previous studies have indicated that increased TSP-1 is involved in the pathological process of fibrosis in multiple organs of DM patients [12, 13], except the retina [14]. However, few reviews have summarized the role of TSP-1 in different chronic diabetic complications. Therefore, this review focuses on the following research questions: is the regulatory mechanism of TSP-1 modulated by hyperglycemia or by DM itself? How do the downstream signaling pathways activated by TSP-1 and the pathogenesis mechanism of fibrosis differ among diabetic organs?

## 2. The Biology of Thrombospondin-1 (TSP-1)

**Figure 1:**
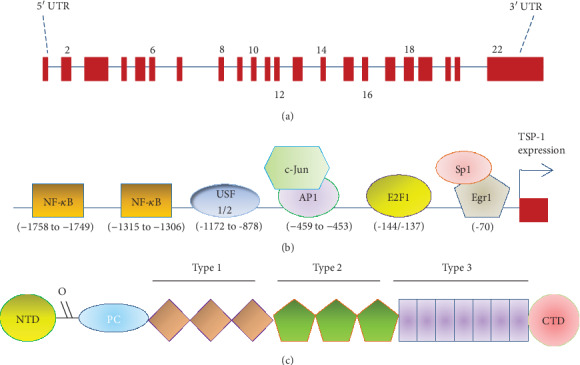
Schematic diagrams of the THBS1 gene and the thrombospondin-1 (TSP-1) protein. (a) Intron-exon organization of the THBS1 gene. The THBS1 gene consists of 16,393 bases including 22 exons. Exons 2-21 encode the 5729 b of mRNA. (b) Different transcription factors bind to the promoter of TSP-1 to induce its expression. USF1/2: upstream stimulatory factors 1/2; Sp1: stimulating protein; NF-*κ*B: nuclear factor-kappa B; Egr1: early growth response 1; and AP1: activator protein 1. (c) Domain organization of TSP-1. NTD: N-terminal domain; O: oligomerization sequence; PC: procollagen homology region; types 1, 2, and 3: the three types of repeat units; CTD: C-terminal domain.

TSP-1 is a common secreted glycoprotein that belongs to the thrombospondin (TSP) family. This protein was named thrombin-sensitive protein (TSP) when it was first identified through its release in response to the activation of platelets by thrombin [15]. According to molecular organization, the thrombospondin gene family, which comprises proteins encoded by five separate genes, is divided into two subfamilies, types A and B [16]. TSP-1 and TSP-2 belong to subgroup A, and subgroup B consists of TSP-3, TSP-4, and TSP-5. TSP-1 is encoded by a gene named Thbs1 (22 exons) in different chromosomal locations among different species [16] (Figure 1(a)). According to previous studies, multiple putative binding sites in the region of the TSP-1 promoter can be activated by hyperglycemia to increase its expression, and some of these TSP-1-stimulating factors include upstream stimulatory factors (USF), activator protein 1 (AP1), E2F1, nuclear factor-kappa B (NF-*κ*B), early growth response 1 (Egr1), and stimulating protein (Sp1) [17–23] (Figure 1(b)). The TSP-1 protein consists of five subunits, namely, an N-terminal domain (NTD), an oligomerization sequence (O), a procollagen homology region (PC), three types of repeat units (types 1, 2, and 3), and a C-terminal domain (CTD) [24] (Figure 1(c)). In contrast to the various structural proteins of the ECM, TSP-1 does not seem to contribute directly to the integrity of a physical entity, such as a fiber or a basement membrane. Rather, it acts contextually to influence cell function by modulating cell-matrix interactions [25]. Although the increased expression of TSP-1 has been proposed to play a critical role in the process of diabetic complications, the identification of such a role has not been fully investigated.

## 3. The Progression of Fibrosis Induced by the Increase of Hyperglycemia-Mediated TSP-1 Expression

**Figure 2:**
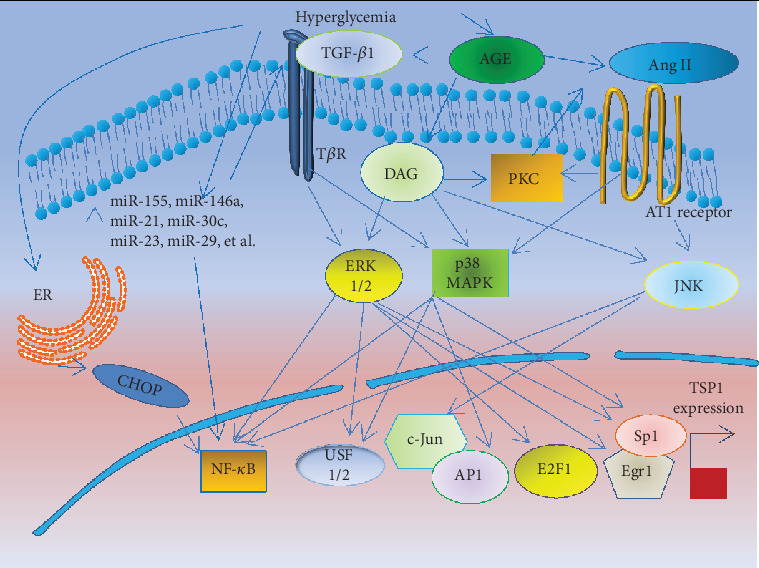
The activation of pathways triggered by hyperglycemia to induce TSP-1 expression. This diagram shows the pathways of PCK and Ang II induced by AGE; in addition, PKC also directly enhances Ang II activity. Then, two pathways induce some transcription factors binding the TSP-1 promoter to enhance the expression of TSP-1 via the MAPK pathway. ER stress and miRNAs also induce the expression of TSP-1 via activating NF-*κ*B signaling. AGE: advanced glycation end products; DAG: diacylglycerol; PKC: protein kinase C; Ang II: angiotensin II; AT1: Ang II type 1; MAPK: mitogen-activated protein kinase; JNK: c-Jun amino terminal kinase; ER: endoplasmic reticulum; ERK: extracellular signal-regulated kinase; USF1/2: upstream stimulatory factors 1/2; Sp1: stimulating protein; NF-*κ*B: nuclear factor-kappa B; Egr1: early growth response 1; AP1: activator protein 1.

**Figure 3:**
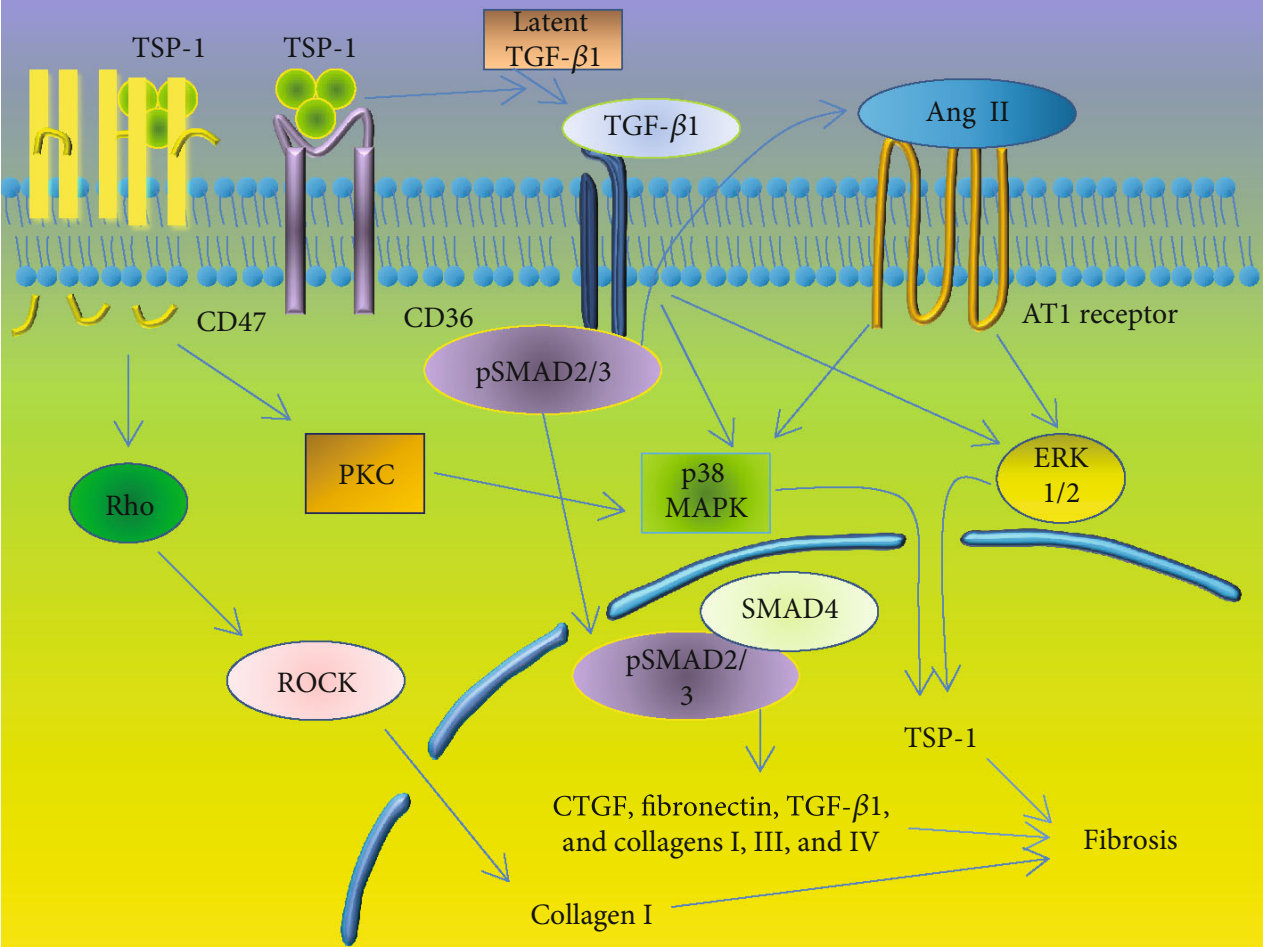
The progression of fibrosis caused by expression of ECM proteins after activation of TSP-1. After binding CD36, TSP-1 activates TGF-*β*1 to initiate cellular fibrosis by triggering two distinct pathways that increase the quantity of expressed ECM proteins. One pathway involves Smad signaling. The receptor-regulated Smad2 and Smad3 are phosphorylated, leading to fibrosis. The other pathway involves non-Smad signals, including the ERK1/2 and MAPK p38 pathways. On the other hand, TSP-1 binding CD47 activates Rho-Rho kinase- (ROCK-) myosin and PCK pathways to enhance TSP-1 expression and TGF-*β*1 activation, ultimately accelerating the progression of fibrosis. TSP-1: thrombospondin-1; PKC: protein kinase C; Ang II: angiotensin II; AT1: Ang II type 1; MAPK: mitogen-activated protein kinase; ROCK: Rho kinase; TGF-*β*1: transforming growth factor *β*1; and CTGF: connective tissue growth factor.

The previous results have shown that hyperglycemia stimulates the expression of TSP-1 [26–28]; however, multiple pathways and/or factors are involved. The entire process is composed of three steps. First, some pathways are triggered by hyperglycemia. Second, these activated pathways induce increased transcription factor activity, and these transcription factors directly bind to the TSP-1 promoter to induce TSP-1 expression (Figure 2). Finally, increased production of TSP-1 leads to binding to different receptors to cause fibrosis by regulating a large proportion of the ECM proteins expressed (Figure 3).

### 3.1. Pathways Activated by Hyperglycemia

#### 3.1.1. Protein Kinase C (PKC)

Protein kinase C (PKC) is a ubiquitous intracellular messenger shown to be required for cell proliferation and migration. In cultured human mesangial cells (MCs), increased PKC activation mimicked the glucose-induced elevation of TSP-1. In contrast, GFX, a PCK inhibitor, abolished the effect of high glucose levels in regulating the expression of TSP-1 [29]. Hence, PKC activity was involved in the mechanism by which glucose stimulated TSP-1 activation, leading to the following question: what is the mechanism by which high glucose levels increase PKC activity? Some authors have suggested that hyperglycemia caused by diabetes induces immediate activation of PKC through the synthesis of diacylglycerol (DAG), which is triggered by oxidative stress [30]. This supposition leads to the following question: what is the precise PKC-activated pathway that induces the expression of TSP-1? It has been shown that the increased expression of TSP-1 induced by PKC is mediated by angiotensin II (Ang II) [31]. On the other hand, PKC can also be activated via the mitogen-activated protein kinase (MAPK) pathway, through which some transcription factors are activated to facilitate TSP-1 expression [32]. This finding confirms that PKC upregulates TSP-1 production via anfractuous signal transduction and complex interactions. Further work is required to identify fully the precise mechanism.

#### 3.1.2. Mitogen-Activated Protein Kinase (MAPK)

The mitogen-activated protein kinase (MAPK) family is a group of serine/threonine kinases that transduce signals from the cell surface to the nucleus, resulting in a diverse range of biological functions by modulating transcription factor function and thereby changing the pattern of gene transcription, including genes related to fibrosis [33]. The MAPK family mainly consists of c-Jun amino terminal kinase (JNK), extracellular signal-regulated kinase (ERK), and MAPK p38. Nakagawa et al. showed that simultaneously blocking both p38 and ERK1/2 resulted in the complete inhibition of TSP-1 expression in rat proximal tubular cells and mouse fibroblasts [34]. This finding was supported by McGillicuddy et al., who demonstrated that blocking the MAPK p38 pathway with the upstream inhibitor SB-203580 also abolished TSP-1 expression [35]. More detailed experiments are necessary to demonstrate that, after activation of the MAPK pathway, TSP-1 expression is still enhanced by the activity of some transcription factors [17, 18, 32, 36–38]. The findings thus far indicate that activation of the MAPK pathway has an additive role in inducing the expression of TSP-1.

#### 3.1.3. Angiotensin II (Ang II)

Angiotensin II (Ang II) is a key effector molecule produced by the renin-angiotensin system (RAS), which is activated in diabetes cases by increased AGE levels [39]. The accumulated evidence suggests that Ang II plays a role in the development of fibrosis through the Ang II type 1 (AT1) receptor, resulting in fibroblast proliferation and net accumulation of fibrillar collagen. A recent report has demonstrated that candesartan is one of the AT1 receptor blockers, which have attracted attention for their potential antifibrotic activity [40]. This attenuation of fibrosis following the blockage of the AT1 receptor is also mediated by impaired activity of transforming growth factor-*β*1 (TGF-*β*1), which is also a factor that induces the expression of TSP-1 [34, 35]. Indeed, it was found that blocking the AT1 receptor antagonist slows the progression of fibrosis associated with diabetic complications by decreasing the expression of TSP-1 [41]. However, blocking the AT2 receptor with PD-123319 does not affect the Ang II-induced upregulation of TSP-1 mRNA or protein, which is induced via the AT1 receptor, not the AT2 receptor. Although it had been demonstrated that Ang II can stimulate the expression of TSP-1 activation in conditioned media, it is not exactly known which signaling pathway(s) downstream of the AT1 receptor mediates this TSP-1 upregulation. However, it has been proven that the MAPK pathway is mediated by the increased TSP-1 expression induced by Ang II. On the other hand, the TSP-1 production induced by Ang II might be associated with MAPK p38 and JNK [42]. Consistent with these studies, Naito found that either the MAPK p38 inhibitor SB-203580 or the JNK inhibitor SP-600125 can significantly reduce TSP-1 production [43]. We believe that TSP-1 is also associated with TGF-*β*1 activity, which plays a critical role, since TGF-*β*1 directly activates the MAPK pathway by elevating some transcription factor activity.

#### 3.1.4. Endoplasmic Reticulum (ER) Stress

Under condition of hyperglycemia, ER stress was activated by increased ROS expression which was produced by glucose oxidation in mitochondria [44]. At the initial stage of ER stress, unfolded protein response (UPR) was activated to enhance the degradation of unfolded proteins [45]. However, if the activation of ER stress is prolonged, fibrosis will be induced due to the acceleration of fibroblast proliferation extracellular matrix protein, such as TGF-*β*1 and TSP-1 [46, 47]. According to a previous study, it has been shown that pharmacological inhibition of ER stress prevents pulmonary fibrosis via suppressing the expression of TGF-*β*1 and TSP-1 [48]. Although the underlying mechanism was not completely revealed yet, some studies have shown that interference or knockout of transcription factor C/EBP homologous protein (CHOP) could significantly reduce the expression of TGF-*β*1 [49, 50]. CHOP is a proapoptotic transcription factor which was activated via three pathways, including protein kinase RNA- (PKR-) like/pancreatic ER kinase (PERK), activating transcription factor 6 (ATF-6), and inositol-requiring enzyme 1 (IRE-1) pathways [51]. In addition to inducing apoptosis, CHOP also plays an important role in modulating NF-*κ*B signaling [52] which is a major regulator to modulate the expression of TSP-1 [21]. Furthermore, an increase in ROS production under ER stress could also activate multiple proinflammatory cytokines, including NF-*κ*B [53]. Therefore, we hypothesized that ER stress induces the expression of TSP-1 via activating NF-*κ*B signaling.

#### 3.1.5. MicroRNAs (miRNAs)

MicroRNAs (miRNAs) are small, noncoding RNA molecules that modulate the gene expression and protein synthesis [54]. In recent years, some reports showed that miRNAs were involved in the fibrosis process of diabetic complications [55–57]. Silambarasan et al. found that more than 100 miRNAs showed differential expression from diabetes patients [58], including miR-155 and miR-146a which play an important role in the process of fibrosis [59]. Although the underlying mechanism was not classified, it was shown that these two miRNAs could activate the expression levels of TGF-*β*1 and NF-*κ*B in renal tissue of diabetic patients and animal models [60]. Furthermore, miR-21 was also increased under the condition of hyperglycemia and targeted to the expression of TSP-1 [61] which was linked to activation of TGF-*β*1/Smad7 signaling [62]. In contrast, other miRNAs were reduced in diabetic conditions, such as miR-30c, miR-23, and miR-29 [63]. These miRNAs could significantly inhibit TGF-*β*1-induced EMT formation [64]. Although there are a large number of miRNAs involved in the fibrosis of diabetic complications [65, 66], the specific mechanisms need to be investigated.

### 3.2. Transcription Factors Involved in the Induction of TSP-1 Expression

The three major pathways discussed above target some transcription factors to promote the expression of TSP-1 after binding to the TSP-1 promoter, including upstream stimulatory factors (USFs) [17, 19], activator protein 1 (AP1) [18, 21], nuclear factor-kappa B (NF-*κ*B) [21], E2F1 [20], Egr1 [22, 67], and Sp1 [22]. In Section 3.2.1–3.2.6, we discuss some primary transcription factors that bind the TSP-1 promoter region to increase TSP-1 transcription upon activation by hyperglycemia.

#### 3.2.1. Upstream Stimulatory Factors (USFs)

Upstream stimulatory factors (USFs), which are encoded by two distinct genes (USF1 and USF2), belong to the basic helix-loop-helix leucine zipper (b-HLH-LZ) family of transcription factors [68]. USF1 and 2 are clearly upregulated by high glucose levels [19]. Activation of PKC under high glucose conditions is critical to the signaling pathway affecting USF1 levels; on the other hand, USF2 appears to be regulated by several signaling pathways, including ERK1/2, MAPK p38, and Ang II [17, 69]. Therefore, the molecular mechanisms governing USF2 synthesis in response to glucose level are involved with multiple pathways. According to a previous study, increased amounts of USF1 and USF2 are bound to the endogenous TSP-1 promoter (-1172 to -878) to regulate hyperglycemia-induced TSP-1 expression [17, 19]. On the other hand, USF1 and USF2 are also involved in the mediated glucose-induced stimulation of TGF-*β*1 [70]. These results indicate that both USF1 and USF2 are important regulators of glucose-induced TSP-1 and may contribute to the development of fibrosis during the progression of diabetic complications.

#### 3.2.2. Activator Protein 1 (AP1)

Activator protein 1 (AP1), as a complex of Jun and other phosphoproteins, belongs to the basic leucine-zipper family of transcription factors. It has been demonstrated that the activity of AP1 is triggered by high glucose levels [18, 19]. It has also been demonstrated that, through this glucose-mediated activity, AP1 binds to the promoter for TSP-1 (from -459 to -453), an interaction enhanced by c-Jun and nuclear proteins [18, 21]. MAPK p38 contributes to increased AP1 expression and binding activity [18].

#### 3.2.3. E2F1

The transcription factor E2F1 is involved in cell cycle regulation by mediating the expression of a large number of genes [71]. According to a previous study, hyperglycemia-induced cell proliferation was mediated by an increase in E2F1 activity [72]; however, this increase in E2F1 activity was not induced by Ang II but was induced by PKC and ERK1/2 [32, 72]. In addition, a recent study showed that E2F1 could also directly bind to the TSP-1 promoter region from -144 to -137. Furthermore, knocking down endogenous E2F1 significantly inhibited TSP-1 promoter activity [20]. Therefore, high levels of E2F-1 activity also contribute to an increase in TSP-1 expression.

#### 3.2.4. Nuclear Factor-Kappa B (NF-*κ*B)

Nuclear factor-kappa B (NF-*κ*B) is an inducible transcription factor consisting of DNA-binding dimers of various subunit combinations that determine functionality. The DNA-binding activity of NF-*κ*B was increased in an animal diabetes model, in which it may have been mediated by ERK1/2, MAPK p38, and/or JNK [36, 73]. Yang et al. demonstrated that there are at least two binding sites for NF-*κ*B (from -1315 to -1306 and from -1758 to -1749) in the TSP-1 promoter region [21]. Indeed, TSP-1 was upregulated in response to the increase in NF-*κ*B activity [74], while inhibition of NF-*κ*B activity reduced TSP-1 protein release and mRNA expression [75]. Although direct evidence of increased NF-*κ*B-dependent TSP-1 expression induced by hyperglycemia has not yet been reported, we believe that NF-*κ*B plays a critical role in the induction of TSP-1 in diabetes. Therefore, we want to determine the role of NF-*κ*B in a future study.

#### 3.2.5. Early Growth Response 1 (Egr1)

Early growth response 1 (Egr1) is an immediately and early activated gene that encodes a protein that binds at approximately -70 in the TSP-1 promoter [76]. Only a sustained increase in glucose flux-induced activation of Egr1 has been reported [77]; however, the exact mechanism by which increased Egr1 activity is induced by hyperglycemia has not been fully investigated. Although a recent study showed that Egr1 expression is increased by the activation of ERK1/2, but not JNK or MAPK p38 [38], more investigation is necessary to determine whether another factor may be involved in the increased activity of Egr1.

#### 3.2.6. Stimulating Protein (Sp1)

Stimulating protein (Sp1) belongs to the two-cysteine–two-histidine zinc-finger (Cys2His2 zinc-finger) family of ubiquitous transcription factors that are mainly involved in basal promoter activity and cell cycle regulation [19]. It has been demonstrated that Sp1 regulates the expression of a wide variety of many ECM genes, such as TGF-*β*1 and TSP-1; however, the role of Sp1 in glucose-mediated gene expression has not been defined [19, 23]. Okamoto et al. found that Sp1 can bind at -267 and -71 in the 5′ region of the TSP-1 gene, which has two GC boxes, to enhance the expression of TSP-1 [23]. Therefore, Sp1 is thought to cooperate with other transcription factors, such as Egr1, which binds at -70, to regulate TSP-1 transcription [22]. MAPK p38 and ERK1/2 all contribute to the increased Sp1 activity that is induced by hyperglycemia [37, 78]; however, no study has reported whether the increased Sp1 activity induced by these pathways to mediate TSP-1 expression is the result of direct binding.

### 3.3. The Downstream Pathway of TSP-1 That Activates ECM Expression

As indicated in the discussion above, multiple pathways and transcription factors induce TSP-1 expression. After TSP-1 expression is increased, TSP-1 binds to some receptors to trigger the expression of ECM proteins that induce fibrosis (Figure 3).

#### 3.3.1. Transforming Growth Factor-*β*1 (TGF-*β*1)

The transforming growth factor-*β* (TGF-*β*) superfamily plays a critical role in promoting the process of fibrosis [79]. According to ligands that are generated by cleavage of a prodomain, the family is divided into TGF-*β*1 and TGF-*β*2. It has been shown that hyperglycemia stimulates an increase in TGF-*β*1 activity, resulting in the promotion of fibrosis in different organs of animal diabetes models [34, 70, 80, 81]. According to previous studies, distinct pathways are thought to be involved in activating TGF-*β*1, including Ang II, MAPK, and TSP-1 [34, 40, 82]. It has been proven that TSP-1 is critical for the activation of TGF-*β*1 in cells exposed to high concentrations of glucose, which contributes to the accumulation of extracellular matrix proteins [34, 83]. This process is needed for CD36 expression [84]. Increasing evidence has shown that the increase in the expression of TGF-*β*1, as induced by elevated levels of glucose, is attenuated after inhibition of TSP-1 with siRNA technology [85].

After activation, TGF-*β*1 initiates a cellular response by binding to its distinct receptor, TGF-*β* receptor II (T*β*RII), to enhance the transcription of 60 ECM-related genes [86]. After ligand binding, two different pathways initiate activation. One is Smad signaling. According to a previous study, T*β*RII activates T*β*RI kinase, which phosphorylates receptor-regulated Smad2 and Smad3 to induce fibrosis [82, 85]. Smad signaling is also critical to a pathway in the process of fibrosis that is induced by other profibrotic factors, such as Ang II and advanced glycation end products (AGE), which are enzymes that generate Ang II [40, 82]. In addition, TGF-*β*1 and Ang II are stress-response proteins, and both are known to be general inducers of fibrosis that are reciprocally activated [82]. Therefore, TGF-*β*1 also enhances the expression of Ang II via Smad signaling. After phosphorylation, Smad2 and Smad3 are translocated from the cytoplasm into the nucleus, where they promote expression of connective tissue growth factor (CTGF), which is a downstream mediator of TGF-*β*1 and plays an essential role in fibrosis [82]. In addition to CTGF, TGF-*β*1 is a potent stimulus for the production of matrix molecules such as collagen I and fibronectin through the Smad pathway [87, 88]. It has been well established that collagen accumulation is a key symptom of fibrosis; in addition, collagen I, and other types of collagen, such as type III and type IV, are induced by TGF-*β*1 [89]. Other pathways initiated through TGF-*β* binding to T*β*RII involve non-Smad signals and include the ERK1/2 and MAPK p38 pathways [90]. The initiation of the non-Smad signaling pathways is very interesting since, as we discuss above, the MAPK family can also induce TSP-1 expression [34]; these findings indicate that TSP-1 is not only an activator of TGF-*β*1 but also the subject of TGF-*β*1 regulation [34, 35, 91]. Thus, a feedback cycle has been established for TSP-1 and TGF-*β*1.

#### 3.3.2. CD47

CD47, originally named integrin-associated protein (IAP), is a receptor of TSP-1 [92]. After binding with the C-terminal domain of TSP-1, CD47 initiates heterotrimeric G-protein signaling that augments the functions of the integrin beta 1, beta 2, and beta 3 families [92–94]. A number of important roles for CD47 have been defined in modulating a range of cellular activities, including cell migration, proliferation, platelet activation, and cellular motility [93]. The CD47 fragment binding to TSP-1 activates the Rho-Rho kinase- (ROCK-) myosin pathway to induce defenestration, which is a pathological dedifferentiation process that leads to fibrosis [92]. In addition, intravenous administration of the ROCK inhibitor Y-23763 (Ycmpd) has been shown to prevent fibrosis [95]. Furthermore, TSP-1-CD47 signaling stimulates the production of reactive oxygen species (ROS), which are also factors that accelerate the progression of fibrosis [94].

#### 3.3.3. CD36

CD36 was described nearly 30 years ago as “glycoprotein IV,” and increased synthesis of the CD36 protein has been observed in people with diabetes [96]. CD36 is involved in a variety of biological processes, including lipid metabolism, inflammation, atherosclerosis, and angiogenesis, depending on the nature of the ligand to which it is exposed, since there are multiple proteins that bind to CD36, including TSP-1 [92, 96, 97]. Atherogenesis is highly associated with the process of fibrosis; therefore, increased CD36 expression has been described in diabetes cases and is thought to be associated with the pathogenesis of diabetic complications. CD36 interacts with TSP-1 via the CSVTCG sequence in TSP-1 [97]. After binding, the CD36-TSP-1 complex activates TGF-*β*1 to initiate cellular fibrosis [98]. Yang et al. found that CD36 siRNA has antifibrotic effects via the reduction in the levels of CD36, TGF-*β*1, and even fibronectin, another important fibrotic marker [98]. Therefore, CD36 is an important factor in a pathway that leads to fibrosis.

## 4. Short Summary

From the complexity of the TSP-1 signaling pathway, it is clear to see the importance of TSP-1 in the induction of fibrosis under elevated glucose. Therefore, many transcription factors and pathways are involved in the TSP-1 signaling pathway. However, the interaction of different molecules has not yet been fully investigated, and the number and nature of them are, indeed, very difficult to determine. Therefore, many questions have not yet been answered. For example, it is not clear whether all of the identified transcription factors induced by hyperglycemia can regulate the expression of TSP-1. Which transcription factor(s) or pathway(s) is a contributor(s) to the increase in TSP-1? What kinds of ECM proteins play important roles in fibrosis due to diabetic complications, and which ECM proteins are secreted at high levels after the induction of TSP-1? We hypothesize that the majority of the answers depend on determining the most important transcription factor or pathway in the induction of TSP-1-mediated fibrosis; only after the results are obtained will we be able to selectively choose drugs and other therapeutic treatments with the greatest effectiveness.

## 5. The Effect of TSP-1 on Fibrosis in Diabetic Complications

**Figure 4:**
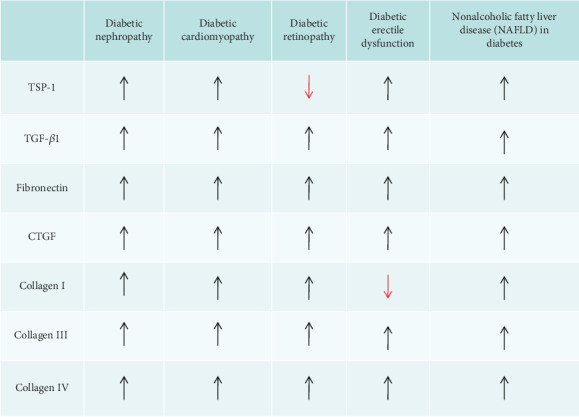
Summary of the expression of TSP-1 and ECM proteins in diabetic complications. TSP-1: thrombospondin-1; TGF-*β*1: transforming growth factor *β*1; CTGF: connective tissue growth factor; ↑: increased expression; ↓: decreased expressions.

As we discuss above, increased TSP-1 expression induced by hyperglycemia is a key regulator of tissue fibrosis in diabetes. Fibrosis, which is characterized by ECM accumulation, is thought to be a common pathological response that leads to diabetic complications and is also a major cause of significant morbidity and mortality of diabetic patients [4, 5]. Although TSP-1 has been shown in different tissues and organs, ECM activation induced by TSP-1 differs qualitatively and quantitatively from tissue to tissue and within various organs (summarized in Figure 4).

### 5.1. The Role of TSP-1 in Diabetic Nephropathy

Diabetic nephropathy (DN) is a common complication in diabetes patients, is marked by ECM accumulation, and is related to the increased production and activity of TGF-*β*1 [24]. It is obvious that TSP-1 converts latent TGF-*β*1 to the active form [34, 99, 100]. Therefore, we also found that TSP-1 and TGF-*β*1 expression was significantly increased in the kidneys of rats in which diabetes was induced by injection of STZ [81]. In addition, compared to wild-type mice, the time course of STZ-induced diabetic nephropathy in TSP-1-null mice was attenuated, as demonstrated by a significant reduction in glomerular matrix accumulation, renal infiltration with inflammatory cells, and renal functional parameter measures [101]. What is the main factor induced by TSP-1 expression in renal tissue? Firstly, three major pathways, including the PKC, MAPK, and Ang II pathways, are involved in triggering TSP-1 expression [17, 34, 41, 43]. Second, miRNAs are involved in increasing the expression of TSP-1. For instance, deregulation of miR-320c, miR-21, and miR-192 which has an effect on TGF-*β*1 and TSP-1 attenuated the progression of DN [62, 63, 66]. Finally, suppression of ER stress could prevent the progression of fibrosis in DN [102], which might be associated with inhibiting the expression of TSP-1 [48]. For ECM expression, each of these pathways has been observed to be involved in diabetic nephropathy, as were the TGF-*β*1 [70], CTGF [103], fibronectin [100], collagen type I [88], and collagen type III and type IV [100] pathways. Therefore, these findings suggest that different types of ECM may be increased to induce fibrosis in DN.

### 5.2. The Role of TSP-1 in Diabetic Cardiomyopathy

Diabetic cardiomyopathy (DC) is characterized by extensive fibrotic changes and expansion of cardiac interstitial areas leading to myocardial hypertrophy and diastolic dysfunction [104]. It was observed that the expression of TSP-1 was upregulated in the myocardium of people in the diabetic group, showing hypertrophy and interstitial fibrosis [105]. On the other hand, a recent report revealed that TSP-1 loss attenuated diabetes mellitus-associated cardiac fibrosis and enhanced myocardial protease activity without significantly enhancing diastolic function in TSP-1-knockout transgenic mice [12]. The hallmarks of DC are increased and include TGF-*β*1, collagen (types I, III, and IV), CTGF, and fibronectin [85, 89, 106]. All of these markers are associated with marked upregulation of TSP-1.

What is the mechanism of unregulated TSP-1 expression and the role of TSP-1 in DC? TSP-1 was found to be a significant mediator of fibrotic complications of diabetes associated with stimulation of Ang II, which is upregulated in diabetes and has been implicated in cardiac fibrosis. This kind of cardiac fibroblast proliferation and the net accumulation of fibrillar collagen are due to increased expression of TSP-1 induced by Ang II [106]. In addition, the activation of the PKC and MAPK pathways were also observed to be enhanced in DC patients [35, 107]. Furthermore, inhibition of ER stress could ameliorate myocardial fibrosis via MAPK in activation in DC [108]. Hence, there are multiple pathways involved in the increased expression of TSP-1 in DC cases. Although some miRNAs were also involved in the fibrosis of DC [109, 110], the specific mechanism needs to be further investigated.

### 5.3. The Role of TSP-1 in Diabetic Retinopathy

Diabetic retinopathy (DR) is a vascular complication of diabetes, and it progresses to the proliferative stage with active neovascularization, which is marked by proliferative diabetic retinopathy (PDR). A clinical marker of DR is the thickening of capillary basement membranes resulting from increased production of collagen types I, III, and IV; fibronectin; CTGF; and TGF-*β*1 [4, 111, 112]. The fibrotic tissue induced by some angiogenic factors tends to contract and leads to retinal detachment. Hence, the relentless abnormal fibrovascular proliferation with subsequent bleeding and retinal detachment results in blindness.

In contrast to the expression of TSP-1 in renal and cardiac tissue, TSP-1 was virtually absent in samples prepared from the eyes of diabetic rats [113]. This result is consistent with our research results showing that TSP-1 is significantly decreased in retinal tissue from diabetic rats compared to that from control rats [114]. It is quite interesting that most ECM proteins are significantly increased [4, 111]; however, these increases are not mediated by TSP-1. On the basis of the literature, TSP-1 has antiangiogenic effects in addition to its function in fibrosis [113]. In contrast to those in macrovascular cells, TSP-1 protein levels are dramatically decreased in response to high glucose levels in microvascular endothelial cells and retinal pigment epithelial cells (RPEs) [115]. However, no exact mechanism has been found to explain this outcome, which may be dependent on cell type- and tissue-specific production of angiogenesis regulators [116]. In DR patients, the antiangiogenic activity of TSP-1 is predominant in the retinal region; therefore, the process of fibrosis is mainly induced by active neovascularization and angiogenesis [4, 113]. VEGF, a major angiogenic factor that contributes to neovascularization of the retina, has been reported to be increased in the presence of diabetes [113, 117]. Some studies have reported dynamic changes in the expression of TSP-1 and VEGF in cases of ischemia-induced retinal neovascularization. An early increase in the expression of VEGF enables vascular endothelial cell migration and proliferation, which later inhibits the enhanced production of TSP-1 [113]. Some miRNAs, such as miR-27b and miR-320a, which are implicated in angiogenesis could suppress the expression of TSP-1 in DR [118]. In the future, more attention should be paid to determining the mechanism by which TSP-1 expression is altered in retinal tissue compared to its expression in other tissues and organs.

### 5.4. The Role of TSP-1 in Diabetic Erectile Dysfunction

Erectile dysfunction (ED) is one of the secondary complications of diabetes, affecting more than 37% of men with DM [119]. The mechanisms of diabetes-related ED are multifactorial, and different types of cells are affected, including endothelial and smooth muscle cells, and the thickness of collagen bundles and proliferation of the intercellular matrix are increased [120]. It has been shown that significantly high levels of oxidative stress and low levels of antioxidants in penile tissue seem to contribute to the increased collagen deposition and fibrosis of the erectile tissue in STZ-treated rats [121]; however, the exact mechanism of diabetic ED remains unknown.

Recently, much attention has been focused on the role of TGF-*β*1 as a fibrogenic cytokine that induces fibrosis, and it has been consistently identified in penile-localized fibrosis [122]. Results from our recent study indicated that TSP-1 expression is, indeed, unregulated in the cavernous tissue of rats with STZ-induced diabetes. From these results, we concluded that an increase in the activation of TSP-1 and TGF-*β*1 under hyperglycemic conditions results in thickening of the tunica albuginea, injury to the collagen fibrils of the penis, and degeneration of smooth muscle cells. These are linked, in turn, to the pathogenesis of diabetes-associated ED [80]. According to previous studies, CTGF, fibronectin, and collagen types III and IV induced by TGF-*β*1 were increased in the corpus cavernosum [86, 123]; however, collagen I and the ratio of collagen I/III fibers were reduced significantly during the decline in penile vascular response [86, 124]. These results might be associated with the reduction in cavernous smooth muscle and endothelial cell content [123]. Although no other recent papers have directly reported on the expression of TSP-1 in the penis, except our study [80], we believe that TSP-1 plays a critical role in the progression of fibrosis in the diabetic penis. Meanwhile, ER stress also plays an important role in the progression of DR through activating inflammation response [125]. Some antifibrotic microRNAs (miR-let7b and miR-let7c) could decrease fibrosis of corpus cavernosum and ameliorate erectile function in diabetic rats [126]; however, the molecular mechanism is not fully clarified.

### 5.5. The Role of TSP-1 in Nonalcoholic Fatty Liver Disease (NAFLD) in Diabetes

Nonalcoholic fatty liver disease (NAFLD) is a common liver disease in diabetes that is also characterized by advanced fibrosis [4, 127]. Fibrosis markers, including TGF-*β*1; collagen types I, III, and IV; CTGF; and fibronectin, are all observed in the diabetic liver tissue [128–130]. Furthermore, significant upregulation in the expression of TSP-1 is also observed in hepatic cells [128]; however, no direct evidence on changes to the expression of TSP-1 in the liver tissue of a diabetes model has been reported. We also believe that TSP-1 plays a critical role in fibrosis in the NAFLD in diabetes models since PKC, MAPK, Ang II, and ER stress [39, 131], which are traditionally considered factors that induce TSP-1 expression, are all activated in the liver tissue after high glucose stimulation. Furthermore, the expression of hepatic microRNAs was altered in NAFLD-related fibrosis [132]. Therefore, future studies should focus on examining the expression of TSP-1 in liver tissue and investigating its specific role in fibrosis.

### 5.6. The Role of TSP-1 in Abnormal Angiogenesis in Diabetes Mellitus

It is well known that diabetic hyperglycemia has been affected in the progression of angiogenesis since elevated blood glucose could lead to integrity loss of capillaries and dysfunctional endothelial cells [133]. As discussed above, the antiangiogenic effects of TSP-1 are implicated in the development of vascular diabetic complications [101, 113, 134], which might be associated with the inhibition of endothelial cell growth [135]. On the cellular level, the endothelial cell is a determinant of initiation of angiogenesis through expressing vascular endothelial cell growth factor (VEGF). However, TSP-1 binds directly to VEGF to inhibit VEGF release [136]; meanwhile, TSP-1 has also been shown to inhibit VEGF signal transduction via decreasing VEGFR2 phosphorylation [137]. Furthermore, the binding of TSP-1 to CD36 results in the suppression of angiogenesis by inhibiting endothelial cell migration and inducing endothelial cell apoptosis [138]. Finally, inhibition of nitric oxide (NO) by TSP-1 has also been shown to inhibit angiogenesis since NO has an effect on endothelial cell proliferation [139]. Therefore, the molecular regulation of angiogenesis by TSP-1 is complicated.

## 6. Summary and Perspectives

Although existing knowledge on the properties of TSP-1 remains incomplete, TSP-1 clearly has an important role in the progression of fibrosis in diabetic complications. In this review, we emphasize that the role of TSP-1 may be different in altered tissues and organs. It is well known that the expression of TSP-1 is not solely associated with many proteins, as it can also stimulate different substances to accelerate the progression of fibrosis. Multiple pathways and ECM proteins have been shown to trigger the progression of fibrosis resulting from increases in TSP-1 expression, as explained in the discussion above. Therefore, anti-TSP-1 agents that reduce TSP-1 expression are definitely effective targets for attenuating the symptoms of fibrosis. Only when all the mechanisms are clearly determined can we understand how and when to abolish TSP-1 expression to protect against fibrosis in diabetic complications.

## References

[1] Xu Y., Wang L., He J. (2013). Prevalence and control of diabetes in Chinese adults. *JAMA*.

[2] Kelly A., Moran A. (2013). Update on cystic fibrosis-related diabetes. *Journal of Cystic Fibrosis*.

[3] Alves Cde A., Aguiar R. A., Alves A. C., Santana M. A. (2007). Diabetes mellitus in patients with cystic fibrosis. *Jornal Brasileiro de Pneumologia*.

[4] Ban C. R., Twigg S. M. (2008). Fibrosis in diabetes complications: pathogenic mechanisms and circulating and urinary markers. *Vascular Health and Risk Management*.

[5] Shah A., Kanaya A. M. (2014). Diabetes and associated complications in the South Asian population. *Current Cardiology Reports*.

[6] Ma T., Dong L. J., Du X. L., Niu R., Hu B. J. (2018). Research progress on the role of connective tissue growth factor in fibrosis of diabetic retinopathy. *International Journal of Ophthalmology*.

[7] Russo I., Frangogiannis N. G. (2016). Diabetes-associated cardiac fibrosis: cellular effectors, molecular mechanisms and therapeutic opportunities. *Journal of Molecular and Cellular Cardiology*.

[8] Tang L., Wu Y., Tian M. (2017). Dapagliflozin slows the progression of the renal and liver fibrosis associated with type 2 diabetes. *American Journal of Physiology. Endocrinology and Metabolism*.

[9] Cui K., Ruan Y., Wang T. (2017). FTY720 supplementation partially improves erectile dysfunction in rats with streptozotocin-induced type 1 diabetes through inhibition of endothelial dysfunction and corporal fibrosis. *The Journal of Sexual Medicine*.

[10] Bornstein P. (2009). Matricellular proteins: an overview. *Journal of Cell Communication and Signaling*.

[11] Maier K. G., Han X., Sadowitz B., Gentile K. L., Middleton F. A., Gahtan V. (2010). Thrombospondin-1: a proatherosclerotic protein augmented by hyperglycemia. *Journal of Vascular Surgery*.

[12] Gonzalez-Quesada C., Cavalera M., Biernacka A. (2013). Thrombospondin-1 induction in the diabetic myocardium stabilizes the cardiac matrix in addition to promoting vascular rarefaction through angiopoietin-2 upregulation. *Circulation Research*.

[13] Murphy-Ullrich J. E., Suto M. J. (2018). Thrombospondin-1 regulation of latent TGF-*β* activation: a therapeutic target for fibrotic disease. *Matrix Biology*.

[14] Sorenson Shoujian Wang C. M., Sheibani R. G. N. (2013). Thrombospondin-1 deficiency exacerbates the pathogenesis of diabetic retinopathy. *Journal of Diabetes & Metabolism*.

[15] Baenziger N. L., Brodie G. N., Majerus P. W. (1972). Isolation and properties of a thrombin-sensitive protein of human platelets. *The Journal of Biological Chemistry*.

[16] Adams J. C., Lawler J. (2011). The thrombospondins. *Cold Spring Harb Perspect Biol*.

[17] Wang S., Skorczewski J., Feng X., Mei L., Murphy-Ullrich J. E. (2004). Glucose up-regulates thrombospondin 1 gene transcription and transforming growth factor-beta activity through antagonism of cGMP-dependent protein kinase repression via upstream stimulatory factor 2. *The Journal of Biological Chemistry*.

[18] Kim S. A., Um S. J., Kang J. H., Hong K. J. (2001). Expression of thrombospondin-1 in human hepatocarcinoma cell lines and its regulation by transcription factor Jun/AP-1. *Molecular and Cellular Biochemistry*.

[19] Sanchez A. P., Sharma K. (2009). Transcription factors in the pathogenesis of diabetic nephropathy. *Expert Reviews in Molecular Medicine*.

[20] Ji W., Zhang W., Xiao W. (2010). E2F-1 directly regulates thrombospondin 1 expression. *PLoS One*.

[21] Yang Y. L., Chuang L. Y., Guh J. Y. (2004). Thrombospondin-1 mediates distal tubule hypertrophy induced by glycated albumin. *The Biochemical Journal*.

[22] Shingu T., Bornstein P. (1994). Overlapping Egr-1 and Sp1 sites function in the regulation of transcription of the mouse thrombospondin 1 gene. *The Journal of Biological Chemistry*.

[23] Okamoto M., Ono M., Uchiumi T. (2002). Up-regulation of thrombospondin-1 gene by epidermal growth factor and transforming growth factor beta in human cancer cells—transcriptional activation and messenger RNA stabilization. *Biochimica et Biophysica Acta*.

[24] Adams J. C. (2001). Thrombospondins: multifunctional regulators of cell interactions. *Annual Review of Cell and Developmental Biology*.

[25] Roberts D. D., Miller T. W., Rogers N. M., Yao M., Isenberg J. S. (2012). The matricellular protein thrombospondin-1 globally regulates cardiovascular function and responses to stress via CD47. *Matrix Biology*.

[26] Matsuo Y., Tanaka M., Yamakage H. (2015). Thrombospondin 1 as a novel biological marker of obesity and metabolic syndrome. *Metabolism*.

[27] Ganguly R., Sahu S., Ohanyan V. (2017). Oral chromium picolinate impedes hyperglycemia-induced atherosclerosis and inhibits proatherogenic protein TSP-1 expression in STZ-induced type 1 diabetic ApoE^−/−^ mice. *Scientific Reports*.

[28] Aiken J., Mandel E. R., Riddell M. C., Birot O. (2018). Hyperglycaemia correlates with skeletal muscle capillary regression and is associated with alterations in the murine double minute-2/forkhead box O1/thrombospondin-1 pathway in type 1 diabetic BioBreeding rats. *Diabetes & Vascular Disease Research*.

[29] Tada H., Kuboki K., Nomura K., Inokuchi T. (2001). High glucose levels enhance TGF-beta1-thrombospondin-1 pathway in cultured human mesangial cells via mechanisms dependent on glucose-induced PKC activation. *Journal of Diabetes and its Complications*.

[30] Kawanami D., Matoba K., Utsunomiya K. (2016). Signaling pathways in diabetic nephropathy. *Histology and Histopathology*.

[31] Ikehara K., Tada H., Kuboki K., Inokuchi T. (2003). Role of protein kinase C-angiotensin II pathway for extracellular matrix production in cultured human mesangial cells exposed to high glucose levels. *Diabetes Research and Clinical Practice*.

[32] Pintus G., Tadolini B., Posadino A. M. (2003). PKC/Raf/MEK/ERK signaling pathway modulates native-LDL-induced E2F-1 gene expression and endothelial cell proliferation. *Cardiovascular Research*.

[33] Ma F. Y., Liu J., Nikolic-Paterson D. J. (2009). The role of stress-activated protein kinase signaling in renal pathophysiology. *Brazilian Journal of Medical and Biological Research*.

[34] Nakagawa T., Lan H. Y., Glushakova O. (2005). Role of ERK1/2 and p38 mitogen-activated protein kinases in the regulation of thrombospondin-1 by TGF-*β*1 in rat proximal tubular cells and mouse fibroblasts. *Journal of the American Society of Nephrology*.

[35] McGillicuddy F. C., O'Toole D., Hickey J. A., Gallagher W. M., Dawson K. A., Keenan A. K. (2006). TGF-beta1-induced thrombospondin-1 expression through the p38 MAPK pathway is abolished by fluvastatin in human coronary artery smooth muscle cells. *Vascular Pharmacology*.

[36] Hsieh S. C., Tsai J. P., Yang S. F., Tang M. J., Hsieh Y. H. (2014). Metformin inhibits the invasion of human hepatocellular carcinoma cells and enhances the chemosensitivity to sorafenib through a downregulation of the ERK/JNK-mediated NF-*κ*B-dependent pathway that reduces uPA and MMP-9 expression. *Amino Acids*.

[37] Murao K., Yu X., Imachi H. (2008). Hyperglycemia suppresses hepatic scavenger receptor class B type I expression. *American Journal of Physiology. Endocrinology and Metabolism*.

[38] Lin K., Fang S., Cai B. (2014). ERK/Egr-1 signaling pathway is involved in CysLT2 receptor-mediated IL-8 production in HEK293 cells. *European Journal of Cell Biology*.

[39] Sipal S., Halici Z., Kiki I. (2012). Comparative study of three angiotensin II type 1 receptor antagonists in preventing liver fibrosis in diabetic rats: stereology, histopathology, and electron microscopy. *Journal of Molecular Histology*.

[40] Okazaki M., Fushida S., Harada S. (2014). The angiotensin II type 1 receptor blocker candesartan suppresses proliferation and fibrosis in gastric cancer. *Cancer Letters*.

[41] Wolf G., Ritz E. (2005). Combination therapy with ACE inhibitors and angiotensin II receptor blockers to halt progression of chronic renal disease: pathophysiology and indications. *Kidney International*.

[42] Kyriakis J. M., Avruch J. (1996). Sounding the alarm: protein kinase cascades activated by stress and inflammation. *The Journal of Biological Chemistry*.

[43] Naito T., Masaki T., Nikolic-Paterson D. J., Tanji C., Yorioka N., Kohno N. (2004). Angiotensin II induces thrombospondin-1 production in human mesangial cells via p38 MAPK and JNK: a mechanism for activation of latent TGF-*β*1. *American Journal of Physiology. Renal Physiology*.

[44] Gall T., Balla G., Balla J. (2019). Heme, heme oxygenase, and endoplasmic reticulum stress—a new insight into the pathophysiology of vascular diseases. *International Journal of Molecular Sciences*.

[45] Hetz C., Saxena S. (2017). ER stress and the unfolded protein response in neurodegeneration. *Nature Reviews. Neurology*.

[46] Luo T., Kim J. K., Chen B., Abdel-Latif A., Kitakaze M., Yan L. (2015). Attenuation of ER stress prevents post-infarction-induced cardiac rupture and remodeling by modulating both cardiac apoptosis and fibrosis. *Chemico-Biological Interactions*.

[47] Borok Z., Horie M., Flodby P. (2020). Grp78 loss in epithelial progenitors reveals an age-linked role for endoplasmic reticulum stress in pulmonary fibrosis. *American Journal of Respiratory and Critical Care Medicine*.

[48] Shi Z., Xu L., Xie H. (2020). Attenuation of intermittent hypoxia-induced apoptosis and fibrosis in pulmonary tissues via suppression of ER stress activation. *BMC Pulmonary Medicine*.

[49] Zhang M., Guo Y., Fu H. (2015). *Chop* deficiency prevents UUO-induced renal fibrosis by attenuating fibrotic signals originated from Hmgb1/TLR4/NF *κ* B/IL-1 *β* signaling. *Cell Death & Disease*.

[50] Kato M., Wang M., Chen Z. (2016). An endoplasmic reticulum stress-regulated lncRNA hosting a microRNA megacluster induces early features of diabetic nephropathy. *Nature Communications*.

[51] Walter P., Ron D. (2011). The unfolded protein response: from stress pathway to homeostatic regulation. *Science*.

[52] Ye L., Zeng Q., Dai H. (2020). Endoplasmic reticulum stress is involved in ventilator-induced lung injury in mice via the IRE1*α*-TRAF2-NF-*κ*B pathway. *International Immunopharmacology*.

[53] Nakajima S., Kitamura M. (2013). Bidirectional regulation of NF-*κ*B by reactive oxygen species: a role of unfolded protein response. *Free Radical Biology & Medicine*.

[54] Lagos-Quintana M., Rauhut R., Lendeckel W., Tuschl T. (2001). Identification of novel genes coding for small expressed RNAs. *Science*.

[55] McClelland A. D., Herman-Edelstein M., Komers R. (2015). miR-21 promotes renal fibrosis in diabetic nephropathy by targeting PTEN and SMAD7. *Clinical Science*.

[56] Liu S., Li W., Xu M., Huang H., Wang J., Chen X. (2014). Micro-RNA 21 targets dual specific phosphatase 8 to promote collagen synthesis in high glucose-treated primary cardiac fibroblasts. *The Canadian Journal of Cardiology*.

[57] Adi N., Adi J., Lassance-Soares R. M., Kurlansky P., Yu H., Webster K. A. (2016). High protein/fish oil diet prevents hepatic steatosis in NONcNZO10 mice; association with diet/genetics-regulated micro-RNAs. *Journal of Diabetes & Metabolism*.

[58] Silambarasan M., Tan J., Karolina D., Armugam A., Kaur C., Jeyaseelan K. (2016). MicroRNAs in hyperglycemia induced endothelial cell dysfunction. *International Journal of Molecular Sciences*.

[59] Zhang X., Pan A., Jia S. (2019). Cystic fibrosis plasma blunts the immune response to bacterial infection. *American Journal of Respiratory Cell and Molecular Biology*.

[60] Huang Y., Liu Y., Li L. (2014). Involvement of inflammation-related miR-155 and miR-146a in diabetic nephropathy: implications for glomerular endothelial injury. *BMC Nephrology*.

[61] Xu X., Song N., Zhang X. (2017). Renal protection mediated by hypoxia inducible factor-1*α* depends on proangiogenesis function of miR-21 by targeting thrombospondin 1. *Transplantation*.

[62] Liu L., Wang Y., Yan R. (2019). BMP-7 inhibits renal fibrosis in diabetic nephropathy via miR-21 downregulation. *Life Sciences*.

[63] Zeng L. F., Xiao Y., Sun L. (2019). A glimpse of the mechanisms related to renal fibrosis in diabetic nephropathy. *Advances in Experimental Medicine and Biology*.

[64] Zou X. Z., Liu T., Gong Z. C., Hu C. P., Zhang Z. (2017). MicroRNAs-mediated epithelial-mesenchymal transition in fibrotic diseases. *European Journal of Pharmacology*.

[65] Conserva F., Barozzino M., Pesce F. (2019). Urinary miRNA-27b-3p and miRNA-1228-3p correlate with the progression of kidney fibrosis in diabetic nephropathy. *Scientific Reports*.

[66] Delic D., Eisele C., Schmid R. (2016). Urinary exosomal miRNA signature in type II diabetic nephropathy patients. *PLoS One*.

[67] Dabir P., Marinic T. E., Krukovets I., Stenina O. I. (2008). Aryl hydrocarbon receptor is activated by glucose and regulates the thrombospondin-1 gene promoter in endothelial cells. *Circulation Research*.

[68] Galibert M. D., Baron Y. (2010). Identification of specific protein/E-box-containing DNA complexes: lessons from the ubiquitously expressed USF transcription factors of the b-HLH-LZ super family. *Methods in Molecular Biology*.

[69] Shi L., Nikolic D., Liu S., Lu H., Wang S. (2009). Activation of renal renin-angiotensin system in upstream stimulatory factor 2 transgenic mice. *American Journal of Physiology. Renal Physiology*.

[70] Zhu Y., Casado M., Vaulont S., Sharma K. (2005). Role of upstream stimulatory factors in regulation of renal transforming growth factor-beta1. *Diabetes*.

[71] Ertosun M. G., Hapil F. Z., Osman Nidai O. (2016). E2F1 transcription factor and its impact on growth factor and cytokine signaling. *Cytokine & Growth Factor Reviews*.

[72] Fujita N., Furukawa Y., Du J. (2002). Hyperglycemia enhances VSMC proliferation with NF-kappaB activation by angiotensin II and E2F-1 augmentation by growth factors. *Molecular and Cellular Endocrinology*.

[73] Gao P., Wu X., Shui H., Jia R. (2013). Fluvastatin inhibits high glucose-induced nuclear factor kappa B activation in renal tubular epithelial cells. *Journal of Nephrology*.

[74] Lim S., MacIntyre D. A., Lee Y. S. (2012). Nuclear factor kappa B activation occurs in the amnion prior to labour onset and modulates the expression of numerous labour associated genes. *PLoS One*.

[75] Wang H. R., Chen D. L., Zhao M. (2012). C-reactive protein induces interleukin-6 and thrombospondin-1 protein and mRNA expression through activation of nuclear factor-*κ*B in HK-2 cells. *Kidney & Blood Pressure Research*.

[76] Shin S. Y., Ko J., Chang J. S. (2002). Negative regulatory role of overexpression of PLC gamma 1 in the expression of early growth response 1 gene in rat 3Y1 fibroblasts. *The FASEB Journal*.

[77] Vedantham S., Thiagarajan D., Ananthakrishnan R. (2014). Aldose reductase drives hyperacetylation of Egr-1 in hyperglycemia and consequent upregulation of proinflammatory and prothrombotic signals. *Diabetes*.

[78] Puebla C., Farias M., Gonzalez M. (2008). High D-glucose reduces SLC29A1 promoter activity and adenosine transport involving specific protein 1 in human umbilical vein endothelium. *Journal of Cellular Physiology*.

[79] Lichtman M. K., Otero-Vinas M., Falanga V. (2016). Transforming growth factor beta (TGF-*β*) isoforms in wound healing and fibrosis. *Wound Repair and Regeneration*.

[80] Zhang X. M., Shi P. H., Cao S. H., Yu H. J., Azad J., Ling S. C. (2010). Expression changes of transforming growth factor-beta1 and thrombospondin-1 in cavernous tissues of diabetic rats. *Urologia Internationalis*.

[81] Wang X., Yan L., Chen W., Xu L., Zhang X. (2009). The renal protective effects of cilostazol on suppressing pathogenic thrombospondin-1 and transforming growth factor-beta expression in streptozotocin-induced diabetic rats. *The Journal of International Medical Research*.

[82] Akhurst R. J. (2012). The paradoxical TGF-*β* vasculopathies. *Nature Genetics*.

[83] Wahab N. A., Schaefer L., Weston B. S. (2005). Glomerular expression of thrombospondin-1, transforming growth factor beta and connective tissue growth factor at different stages of diabetic nephropathy and their interdependent roles in mesangial response to diabetic stimuli. *Diabetologia*.

[84] Mir F. A., Contreras-Ruiz L., Masli S. (2015). Thrombospondin-1-dependent immune regulation by transforming growth factor-*β*2-exposed antigen-presenting cells. *Immunology*.

[85] Tang M., Zhou F., Zhang W. (2011). The role of thrombospondin-1-mediated TGF-*β*1 on collagen type III synthesis induced by high glucose. *Molecular and Cellular Biochemistry*.

[86] Zhou F., Li G. Y., Gao Z. Z. (2012). The TGF-*β*1/Smad/CTGF pathway and corpus cavernosum fibrous-muscular alterations in rats with streptozotocin-induced diabetes. *Journal of Andrology*.

[87] Sriram S., Robinson P., Pi L., Lewin A. S., Schultz G. (2013). Triple combination of siRNAs targeting TGF*β*1, TGF*β*R2, and CTGF enhances reduction of collagen I and smooth muscle actin in corneal fibroblasts. *Investigative Ophthalmology & Visual Science*.

[88] Khan S., Jena G. (2014). Sodium butyrate, a HDAC inhibitor ameliorates eNOS, iNOS and TGF-*β*1-induced fibrogenesis, apoptosis and DNA damage in the kidney of juvenile diabetic rats. *Food and Chemical Toxicology*.

[89] Forino M., Torregrossa R., Ceol M. (2006). TGF*β*1 induces epithelial-mesenchymal transition, but not myofibroblast transdifferentiation of human kidney tubular epithelial cells in primary culture. *International Journal of Experimental Pathology*.

[90] Wrana J. L. (2013). Signaling by the TGF*β* superfamily. *Cold Spring Harbor Perspectives in Biology*.

[91] Jefferson B., Ali M., Grant S. (2020). Thrombospondin-1 exacerbates acute liver failure and hepatic encephalopathy pathology in mice by activating transforming growth factor *β*1. *The American Journal of Pathology*.

[92] Venkatraman L., Tucker-Kellogg L. (2013). The CD47-binding peptide of thrombospondin-1 induces defenestration of liver sinusoidal endothelial cells. *Liver International*.

[93] Brown E. J., Frazier W. A. (2001). Integrin-associated protein (CD47) and its ligands. *Trends in Cell Biology*.

[94] Rogers N. M., Sharifi-Sanjani M., Csanyi G., Pagano P. J., Isenberg J. S. (2014). Thrombospondin-1 and CD47 regulation of cardiac, pulmonary and vascular responses in health and disease. *Matrix Biology*.

[95] Murata T., Arii S., Mori A., Imamura M. (2003). Therapeutic significance of Y-27632, a rho-kinase inhibitor, on the established liver fibrosis. *The Journal of Surgical Research*.

[96] Zhang D., Zhang R., Liu Y. (2018). CD36 gene variants is associated with type 2 diabetes mellitus through the interaction of obesity in rural Chinese adults. *Gene*.

[97] Asch A. S., Silbiger S., Heimer E., Nachman R. L. (1992). Thrombospondin sequence motif (CSVTCG) is responsible for CD36 binding. *Biochemical and Biophysical Research Communications*.

[98] Yang Y. L., Lin S. H., Chuang L. Y. (2007). CD36 is a novel and potential anti-fibrogenic target in albumin-induced renal proximal tubule fibrosis. *Journal of Cellular Biochemistry*.

[99] Hayashi H., Sakai K., Baba H., Sakai T. (2012). Thrombospondin-1 is a novel negative regulator of liver regeneration after partial hepatectomy through transforming growth factor-beta1 activation in mice. *Hepatology*.

[100] Cui W., Maimaitiyiming H., Qi X., Norman H., Wang S. (2013). Thrombospondin 1 mediates renal dysfunction in a mouse model of high-fat diet-induced obesity. *American Journal of Physiology. Renal Physiology*.

[101] Daniel C., Schaub K., Amann K., Lawler J., Hugo C. (2007). Thrombospondin-1 is an endogenous activator of TGF-*β* in experimental diabetic nephropathy in vivo. *Diabetes*.

[102] Qi W., Mu J., Luo Z. F. (2011). Attenuation of diabetic nephropathy in diabetes rats induced by streptozotocin by regulating the endoplasmic reticulum stress inflammatory response. *Metabolism*.

[103] Dai H. Y., Ma L. N., Cao Y. (2017). Protection of CTGF antibody against diabetic nephropathy in mice via reducing glomerular *β*-catenin expression and podocyte epithelial-mesenchymal transition. *Journal of Cellular Biochemistry*.

[104] Jia G., DeMarco V. G., Sowers J. R. (2016). Insulin resistance and hyperinsulinaemia in diabetic cardiomyopathy. *Nature Reviews. Endocrinology*.

[105] Yu J., Fei J., Azad J., Gong M., Lan Y., Chen G. (2012). Myocardial protection by *Salvia miltiorrhiza* injection in streptozotocin-induced diabetic rats through attenuation of expression of thrombospondin-1 and transforming growth factor-*β*1. *The Journal of International Medical Research*.

[106] Belmadani S., Bernal J., Wei C. C. (2007). A thrombospondin-1 antagonist of transforming growth factor-beta activation blocks cardiomyopathy in rats with diabetes and elevated angiotensin II. *The American Journal of Pathology*.

[107] Soetikno V., Sari F. R., Sukumaran V. (2012). Curcumin prevents diabetic cardiomyopathy in streptozotocin-induced diabetic rats: possible involvement of PKC-MAPK signaling pathway. *European Journal of Pharmaceutical Sciences*.

[108] Lian J., Chen J., Yuan Y. (2017). Cortex Mori Radicis extract attenuates myocardial damages in diabetic rats by regulating ERS. *Biomedicine & Pharmacotherapy*.

[109] Kambis T. N., Shahshahan H. R., Kar S., Yadav S. K., Mishra P. K. (2019). Transgenic expression of miR-133a in the diabetic Akita heart prevents cardiac remodeling and cardiomyopathy. *Frontiers in Cardiovascular Medicine*.

[110] Zhang X., Dong S., Jia Q. (2019). The microRNA in ventricular remodeling: the miR-30 family. *Bioscience Reports*.

[111] Van Geest R. J., Klaassen I., Lesnik-Oberstein S. Y. (2013). Vitreous TIMP-1 levels associate with neovascularization and TGF-*β*2 levels but not with fibrosis in the clinical course of proliferative diabetic retinopathy. *Journal of Cell Communication and Signaling*.

[112] Shanmuganathan S., Angayarkanni N. (2019). Chebulagic acid and chebulinic acid inhibit TGF-*β*1 induced fibrotic changes in the chorio-retinal endothelial cells by inhibiting ERK phosphorylation. *Microvascular Research*.

[113] Sheibani N., Sorenson C. M., Cornelius L. A., Frazier W. A. (2000). Thrombospondin-1, a natural inhibitor of angiogenesis, is present in vitreous and aqueous humor and is modulated by hyperglycemia. *Biochemical and Biophysical Research Communications*.

[114] Zhang H. Y., Wang J. Y., Zhang X. M., Shen F. (2007). The changes of TSP-1 expression in the retina of STZ-induced rat diabetic mellitus model with pioglitazone. *Fen Zi Xi Bao Sheng Wu Xue Bao*.

[115] Huang Q., Sheibani N. (2008). High glucose promotes retinal endothelial cell migration through activation of Src, PI3K/Akt1/eNOS, and ERKs. *American Journal of Physiology. Cell Physiology*.

[116] Bhattacharyya S., Marinic T. E., Krukovets I., Hoppe G., Stenina O. I. (2008). Cell type-specific post-transcriptional regulation of production of the potent antiangiogenic and proatherogenic protein thrombospondin-1 by high glucose. *The Journal of Biological Chemistry*.

[117] Zorena K., Raczyńska D., Raczyńska K. (2013). Biomarkers in diabetic retinopathy and the therapeutic implications. *Mediators of Inflammation*.

[118] Zampetaki A., Willeit P., Burr S. (2016). Angiogenic microRNAs linked to incidence and progression of diabetic retinopathy in type 1 diabetes. *Diabetes*.

[119] Maiorino M. I., Bellastella G., Della Volpe E. (2017). Erectile dysfunction in young men with type 1 diabetes. *International Journal of Impotence Research*.

[120] Gur S., Hellstrom W. J. G. (2019). Harnessing stem cell potential for the treatment of erectile function with diabetes mellitus: from preclinical/clinical perspectives to penile tissue engineering. *Current Stem Cell Research & Therapy*.

[121] Suresh S., Prakash S. (2011). Effect of *Mucuna pruriens* (Linn.) on oxidative stress-induced structural alteration of corpus cavernosum in streptozotocin-induced diabetic rat. *The Journal of Sexual Medicine*.

[122] Gonzalez-Cadavid N. F., Rajfer J. (2005). Mechanisms of disease: new insights into the cellular and molecular pathology of Peyronie’s disease. *Nature Clinical Practice. Urology*.

[123] Zhang L. W., Piao S., Choi M. J. (2008). Role of Increased Penile Expression of Transforming Growth Factor‐*β*1 and Activation of the Smad Signaling Pathway in Erectile Dysfunction in Streptozotocin‐Induced Diabetic Rats. *The Journal of Sexual Medicine*.

[124] Luttrell I. P., Swee M., Starcher B., Parks W. C., Chitaley K. (2008). Erectile dysfunction in the type II diabetic db/db mouse: impaired venoocclusion with altered cavernosal vasoreactivity and matrix. *American Journal of Physiology. Heart and Circulatory Physiology*.

[125] Robles-Rivera R. R., Castellanos-Gonzalez J. A., Olvera-Montano C. (2020). Adjuvant therapies in diabetic retinopathy as an early approach to delay its progression: the importance of oxidative stress and inflammation. *Oxidative Medicine and Cellular Longevity*.

[126] Zhu L. L., Huang X., Yu W., Chen H., Chen Y., Dai Y. T. (2018). Transplantation of adipose tissue-derived stem cell-derived exosomes ameliorates erectile function in diabetic rats. *Andrologia*.

[127] Nakahara T., Japan Study Group of Nonalcoholic Fatty Liver Disease (JSG-NAFLD), Hyogo H. (2014). Type 2 diabetes mellitus is associated with the fibrosis severity in patients with nonalcoholic fatty liver disease in a large retrospective cohort of Japanese patients. *Journal of Gastroenterology*.

[128] Chavez-Tapia N. C., Rosso N., Tiribelli C. (2012). Effect of intracellular lipid accumulation in a new model of non-alcoholic fatty liver disease. *BMC Gastroenterology*.

[129] Lo L., McLennan S. V., Williams P. F. (2011). Diabetes is a progression factor for hepatic fibrosis in a high fat fed mouse obesity model of non-alcoholic steatohepatitis. *Journal of Hepatology*.

[130] Leite N. C., Salles G. F., Cardoso C. R. L., Villela-Nogueira C. A. (2013). Serum biomarkers in type 2 diabetic patients with non-alcoholic steatohepatitis and advanced fibrosis. *Hepatology Research*.

[131] Van Campenhout S., Tilleman L., Lefere S. (2020). Myeloid-specific IRE1alpha deletion reduces tumour development in a diabetic, non-alcoholic steatohepatitis-induced hepatocellular carcinoma mouse model. *Metabolism*.

[132] Leti F., Malenica I., Doshi M. (2015). High-throughput sequencing reveals altered expression of hepatic microRNAs in nonalcoholic fatty liver disease-related fibrosis. *Translational Research*.

[133] Okonkwo U., DiPietro L. (2017). Diabetes and wound angiogenesis. *International Journal of Molecular Sciences*.

[134] Stenina O. I., Krukovets I., Wang K. (2003). Increased expression of thrombospondin-1 in vessel wall of diabetic Zucker rat. *Circulation*.

[135] Lawler P. R., Lawler J. (2012). Molecular basis for the regulation of angiogenesis by thrombospondin-1 and -2. *Cold Spring Harbor Perspectives in Medicine*.

[136] Greenaway J., Lawler J., Moorehead R., Bornstein P., Lamarre J., Petrik J. (2007). Thrombospondin-1 inhibits VEGF levels in the ovary directly by binding and internalization via the low density lipoprotein receptor-related protein-1 (LRP-1). *Journal of Cellular Physiology*.

[137] Zhang X., Kazerounian S., Duquette M. (2009). Thrombospondin-1 modulates vascular endothelial growth factor activity at the receptor level. *The FASEB Journal*.

[138] Jimenez B., Volpert O. V., Crawford S. E., Febbraio M., Silverstein R. L., Bouck N. (2000). Signals leading to apoptosis-dependent inhibition of neovascularization by thrombospondin-1. *Nature Medicine*.

[139] Isenberg J. S., Ridnour L. A., Perruccio E. M., Espey M. G., Wink D. A., Roberts D. D. (2005). Thrombospondin-1 inhibits endothelial cell responses to nitric oxide in a cGMP-dependent manner. *Proceedings of the National Academy of Sciences*.

